# Social Network Analysis As a Tool for Research Policy

**DOI:** 10.1371/journal.pntd.0004266

**Published:** 2015-12-31

**Authors:** Dieter Vanderelst

**Affiliations:** 1 Faculty of Environment and Technology, University of the West of England, Frenchay Campus,Bristol, United Kingdom; 2 Faculty of Applied Economics, University of Antwerp, City Campus, Antwerp, Belgium; Duke University, UNITED STATES

An extensive range of metrics has been proposed to quantify the scientific impact of papers, journals, individual researchers, and institutions [1]. A recent article reviewed no less than 57 metrics used for measuring research output [2]. Nevertheless, new ways of measuring research are still being proposed. A relatively novel method for quantifying research output is Social Network Analysis (SNA) [1,3]. In this context, SNA is a method for mapping and measuring the relationships between papers, journals, researchers, and institutions. SNA allows for the capturing of aspects of scientific impact and importance not picked up by other metrics [3]. As such, metrics derived from SNA have been used as alternatives to complement more established metrics. For example, in an earlier study, Morel et al. [4] suggested nine Brazilian research institutions to be targeted by funding programmes based on SNA. The authors selected institutions interconnecting several fragments of the research network, assuming these would facilitate the exchange of knowledge throughout the network.

In their paper, Bender et al. [5] used SNA to assess national and international collaborations of Germany-based researchers and research institutions working on five neglected tropical diseases (NTDs). Based on their analysis, the authors identified promising researchers and opportunities for strengthening national and international collaborations.

**Fig 1:**
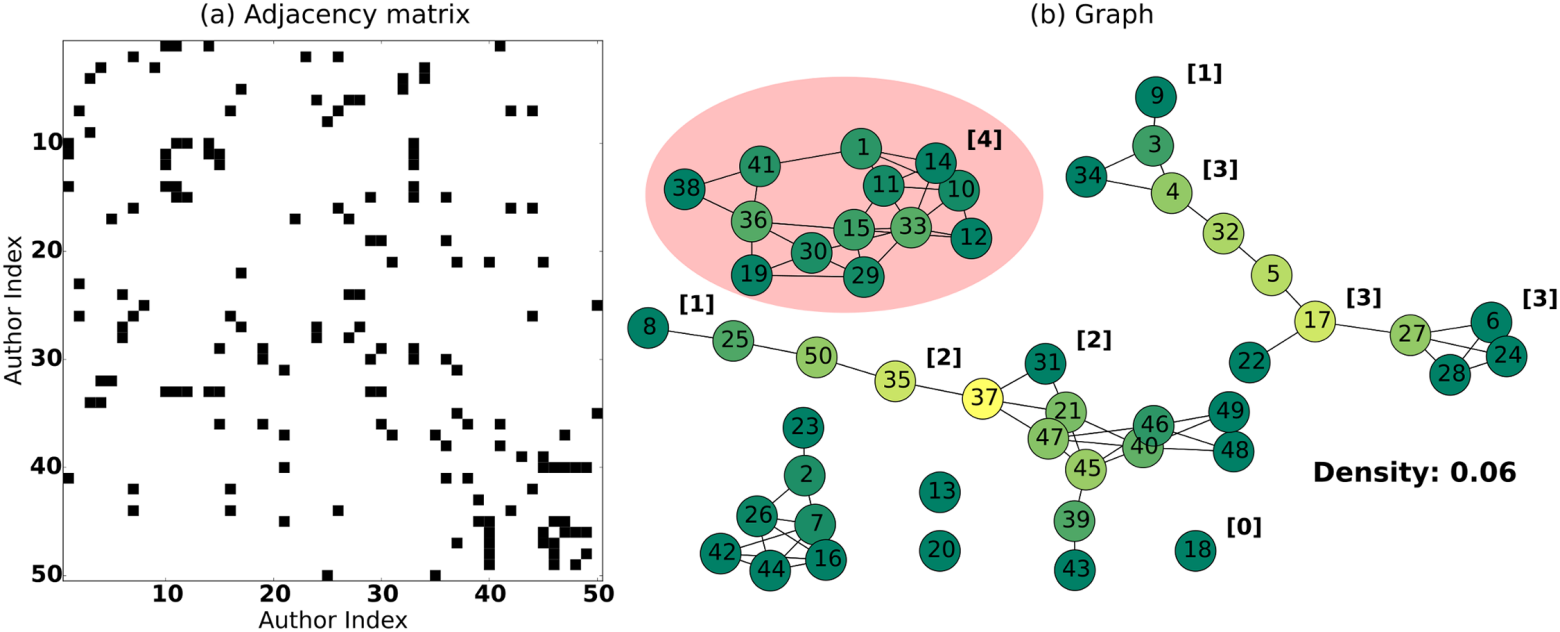
(a) Example of an Adjacency Matrix, the generic data format used in Social Network Analysis. This example represents a network of authors. The rows and columns of the matrix represent 50 different authors. Black squares indicate the author in the row and column have at least co-authored one paper together. Note that the matrix is symmetrical. (b) Graphical representation of the adjacency matrix in panel a. This visualization reveals that the network consists of seven components, one of which has been circled. The colours of the nodes are determined by their betweenness centrality. In this network, author 37 has the highest betweenness centrality. The degree of selected author nodes is indicated using a number in brackets. The density of this network is about 0.06 (i.e., the average value of the adjacency matrix, omitting the diagonal).

In spite of the apparent complexity of network visualizations, the underlying data is simple. SNA is based on a so-called adjacency matrix or connection matrix [6]. In the simplest case, this is a table with the same entries for both rows and columns, i.e., akin to the format of a correlation matrix (see Fig 1A). A given cell in the matrix contains a 1 or a 0 depending on whether the row and column entry are connected or not.

The data used in the paper by Bender et al. consists of ten admittedly large adjacency matrices (provided as supporting material S3–S12 in [5]). For each of five NTDs, an adjacency matrix for both authors and research institutions was constructed. That is, both rows and columns of the adjacency matrices listed either individual researchers or their institutions. A given cell in the matrix contains a 1 if two authors (or institutions) co-authored at least one paper. The data used in constructing the matrices was retrieved from Scopus, a database of academic papers.

From these adjacency matrices, Bender et al. compute a number of metrics. Some SNA metrics are very straightforward to compute. For example, the network density is calculated as the proportion of 1s in the matrix (omitting the diagonal). The degree of an author or institution is given by its number of links in the network and can be calculated by summing the corresponding row or column in the matrix (Fig 1B). Other statistics require more complex calculations. For example, finding groups of authors that are interconnected (either directly or indirectly) but are not connected to other authors, i.e., so-called components (Fig 1B), requires inspecting the matrix using efficient search algorithms. Another metric that requires some computation to calculate is the betweenness centrality. This metric reflects the importance of an author or institution in “holding the network together” (Fig 1B). Removing an author or institution with a high betweenness centrality from the network was likely to result in a more fragmented network, i.e., a network with more components.

Bender et al. found that the author networks are only sparsely connected. They have low-density metrics and consist of many independent components (Table 4 in [5]). What this signifies depends on the assumption one makes about what co-author relationships capture. One could simply interpret the existence of multiple non-connected components as the fact that several groups of authors tend to co-author papers exclusively together. Alternatively, one could, as the authors do, interpret the fragmented structure of the network as reflecting a lack in sharing of knowledge and expertise between the various components. Whether the co-author relationships are a faithful representation of the knowledge exchange across the network is an open question. However, if so, one could speculate about a range of corrective measures to increase the number of co-author connections [4].

From the presented analysis, it transpired that international collaborations of German researchers are mostly restricted to high-income countries (Table 3 and Fig 3 in [5]), in particular the United Kingdom and the United States. The authors reported that the number of co-authorships with partners in low-income countries is only a tenth of the number of those in high-income countries, with very few of those being African partners. These figures, they argued, highlight the need for identifying and addressing factors limiting collaboration with partners in low- and middle-income countries. As a case in point, data pertaining to Brazil indicates that a strategic commitment to support NTD research can result in increased research output [4] and international collaboration (Table 3 in [5]).

Finally, the authors put forward an intriguing suggestion. They proposed that promising authors can be identified by their betweenness centrality (see Fig 1B). The advantage of this measure, they claimed, is that it allows for the identification of more junior researchers that have not yet accumulated sufficient papers in order to be favoured by more classic metrics such as the h-index (Table 4 in [5]). In this, their approach is reminiscent of the suggestion by Morel et al. [4] to support institutes that increase the research network connectivity. As it stands, the value of the betweenness centrality in spotting emerging talent remains to be confirmed by other research. However, their suggestion is interesting in that it prioritizes strengthening and building a resilient, highly connected research network rather than focusing exclusively on highly productive individuals, as in [7].

In summary, the paper of Bender et al. is part of an emerging trend to use Social Network Analysis in formulating research policy recommendations. The presented data give German policymakers access to a set of novel metrics and are likely to be an impetus for further research. However, it should be highlighted that, as indicated above, the paper's significance for policy critically hinges on a number of assumptions about what co-author relationships capture. These assumptions determine which policies can be derived from their analysis. Another reason for caution is that there is currently limited evidence regarding the effectiveness of SNA-based policies in supporting research capacity-building at an (inter)national level. Recognizing the need for caution, Bender et al. do not explicate specific policy recommendations. However, speculating more freely about potential policies, in analogy with the approach of Morel et al., one can surmise that working groups can be established to identify opportunities for collaboration and to set funding priorities. In addition, funding decisions could be based on both classic measures of impact and measures derived from network analysis.
