# Correction: Social Network Analysis As a Tool for Research Policy

**DOI:** 10.1371/journal.pntd.0004410

**Published:** 2016-01-21

**Authors:** Dieter Vanderelst

Reference 6 is written incorrectly. The correct reference is: Bender ME, Edwards S, von Philipsborn P, Steinbeis F, Keil T, Tinnemann P (2015) Using Co-authorship Networks to Map and Analyse Global Neglected Tropical Disease Research with an Affiliation to Germany. PLoS Negl Trop Dis 9(12): e0004182. doi:10.1371/journal.pntd.0004182
